# A small molecular agent YL529 inhibits VEGF-D-induced lymphangiogenesis and metastasis in preclinical tumor models in addition to its known antitumor activities

**DOI:** 10.1186/s12885-015-1451-2

**Published:** 2015-07-18

**Authors:** Youzhi Xu, Wenjie Lu, Peng Yang, Wen Peng, Chunting Wang, Manli Li, Yan Li, Guobo Li, Nana Meng, Hongjun Lin, Lixin Kan, Siying Wang, Shengyong Yang, Luoting Yu, YingLan Zhao

**Affiliations:** 1 Department of Pathophysiology, School of Basic Medicine, Anhui Medical University, 81#, Mei Shan Road, Hefei, 230032 China; 2 State Key Laboratory of Biotherapy and Cancer Center, West China Hospital, West China Medical School, and Collaborative Innovation Center for Biotherapy, Sichuan University, 17#, 3rd Section, Ren min South Road, Chengdu, 610041 China; 3 Department of Transfusion, the First Affiliated Hospital of Anhui Medical University, 81#, Mei Shan Road, Hefei, 230032 China; 4 Department of Oncology, The People’s Hospital of Guizhou Province, 83#, Zhong Shan East Road, Guiyang, 550004 China

**Keywords:** YL529, Lymphangiogenesis, Metastasis, VEGF-D, VEGFR-3

## Abstract

**Background:**

The lymph node metastasis is a key early step of the tumor metastatic process. VEGFD-mediated tumor lymphangiogenesis plays a key role, since down-regulation of p-VEGFR-3 could block the lymph node metastasis. YL529 has been reported to possess potent anti-angiogenesis and antitumor activities; however, its roles in tumor-associated lymphangiogenesis and lymphatic metastasis remain unclear.

**Method:**

We investigated the effect of YL529 on tumor-associated lymphangiogenesis and lymph node metastasis using *in vitro* lymph node metastasis models and *in vivo* subcutaneous tumor models in C57 BL/6 mice.

**Result:**

We found that YL529 inhibited VEGF-D-induced survival, proliferation and tube-formation of Human Lymphatic Endothelial Cells. Furthermore, in established *in vitro* and *in vivo* lymph node metastasis models using VEGF-D-LL/2 cells, YL529 significantly inhibited the tumor-associated lymphangiogenesis and metastasis. At molecular level, YL529 down-regulated p-VEGFR-3, p-JNK and Bax while up-regulated Bcl-2.

**Conclusion:**

YL529 provided the therapeutic benefits by both direct effects on tumor cells and inhibiting lymphangiogenesis and metastasis via the VEGFR-3 signaling pathway, which may have significant direct clinical implications.

## Background

Tumor metastasis is the key and final cause of cancer mortality [1–3]. It usually occurs via the hematogenic and the lymphogenic metastasis routes [4, 5], and the latter is considered to be a very important route contributing to metastasis of solid tumors [6].

Tumor lymphangiogenesis is regulated by many important factors, such as vascular endothelial growth factor (VEGF) and its receptor subtype members (VEGFRs). VEGFR-1 ~ 3 are almost exclusively located on the surface of vascular endothelial cells and lymphatic vessels in normal tissues and are up-regulated only during embryonic and tumor. Among all of VEGFs and VEGFRs, the Vascular endothelial growth factor D (VEGF-D) is indispensable for development of the lymphatic system and VEGFR-3 has also been implicated as the major effectors of lymphangiogenesis and regulates lymphatic vessel growth [1, 7]; VEGF-C and VEGF-D are the ligands for the tyrosine kinases VEGFR-3 and VEGFR 2, which mainly regulates the growth of lymphatic vessels via their receptor VEGFR-3, and partly regulates the growth of blood vessels via VEGFR-2 [8, 9]. Since literatures have showed that tumor-induced lymphangiogenesis driven by VEGF-C and VEGF-D-induced activation of VEGFR-3 could promote regional lymph node metastasis in multiple solid tumors [10–13]. Consistently, tumor cells with up-regulated VEGF-C and VEGF-D could increase the intratumoral and peritumoral lymphangiogenesis and exacerbate metastasis to local lymph nodes and distant organs [14]. And some researchers have reported that VEGF-C plays an important role in the process of lung cancer metastasis, but there are just a few reports about VEGF-D as the ligand for VEGFR-3 in the tumor metastasis process *in vivo* [1, 2]. Therefore, targeting of VEGF-C or VEGF-D/VEGFR-3 could potentially block the lymphatic metastasis.

Current studies suggest that therapeutic strategies targeting tumor lymphangiogenesis via the VEGF/VEGFR kinase axis are promising approaches for the treatment of cancer lymphogenic metastasis. However, quite a few target therapies show toxicity and have only moderate response rates for tumor treatment.

A number of small molecules, which could inhibit the intrinsic tyrosine kinase activity of VEGFR, have been reported previously with a range of nanomolar potencies, specificities, and pharmacokinetic properties [15–17]. Our group also focused on developing a small molecular compound that potently and selectively blocks the VEGF-D/VEGFR3 receptor system after oral administration, suitable for the chronic therapy of VEGF-D/VEGFR3-dependent pathological lymphangiogenesis. We previously described YL529, a small molecular anti-cancer agent synthesized by our laboratory, inhibits tumor neovascularization and cell proliferation in a panel of cell lines and in tumor-bearing mouse models. YL529 has demonstrable potent antitumor and anti-angiogenic properties against human umbilical vein endothelial cells (HUVECs) by blocking VEGF_165_-induced VEGFR-2 autophosphorylation [18]. YL529 also inhibited the phosphorylation of VEGFR-3. However, it is still unclear whether YL529 could effectively inhibit lymphangiogenesis and the associated lung and lymphatic metastasis, or whether YL529 could potentially improve the survival of the affected individuals.

In present study,we choose to detect the expression of VEGF-D in VEGF-D-LL/2 cell after YL529 treatment because other researchers in our group have been successfully established the over-expression VEGF-D in Lewis lung cancer cells VEGF-D-LL/2 cell *in vitro*. And it is true that tumor metastatic models have been successfully established via injecting over-expression VEGF-D Lewis lung cancer cells (VEGF-D-LL/2) in C57 BL/6 mice [19]. Our current study aimed to further address these issues, and we found that YL529 blocked VEGF-D-induced lymphangiogenesis and inhibited tumor growth efficiently at the dosage of 150 mg/kg/day or even less, as a result, affected mice have significantly better survival rate. This novel finding may have significant direct clinical implications.

## Methods

### Preparation of YL529

**Fig. 1:**
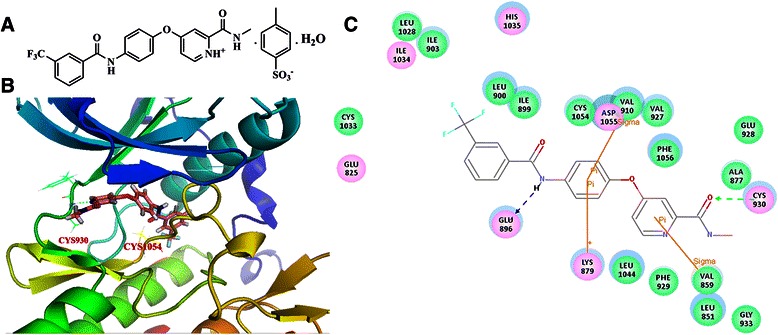
The chemical structure of YL529 and its interaction with VEGFR3. **a** The chemical structure of YL529; **b** and **c** The interactions between YL529 and VEGFR3 were shown in 3-D structure and 2-D map

The route of synthesis of YL529 (N-methyl-4-(4-(3-(trifluoromethyl)benzamido) phenoxy)picolinamide4-methylbenzenesulfonate) was provided by State Key Laboratory of Biotherapy, Sichuan University (Sichuan, China), its structural formula is shown in Fig. 1a, YL529 were analyzed and identified by high performance liquid chromatography (HPLC, Waters, MA, USA) and nuclear magnetic resonance (NMR). For all *in vitro* assays, YL529 was prepared as stock solution in dimethyl-sulphoxide (DMSO) at final DMSO concentration 0.05 % (V/V) and diluted in the cell culture medium. For *in vivo* studies, YL529 was dissolved in ultrapure water with 0.5 % sodium carboxymethylcellulose (CMC-Na) combined with 2 % Tween-20, then administered by oral gavages at 10 ml/kg/day.

### Homology modeling and molecular docking methods

The sequence of human VEGFR3 (Genbank accession number: AAO89505.1) was extracted from the NCBI protein sequence database. The crystal structure of VEGFR2 (PDB code: 4ASD) was used as a template for human VEGFR3 homology modeling; the sequence identity between VEGFR2 and VEGFR3 is larger than 70 %. The homology model of VEGFR3 was generated using the MODELER program implemented in Discovery Studio (DS) 3.1 software package.

The established VEGFR3 homology model was then used for the following docking study. A sphere containing the key residues in VEGFR3 (including CYS930, ALA877, VAL859, PHE929, LEU1044, LYS879, ASP1055, CYS1054, VAL910, GLU896, ILE899, and LEU900) was defined as the binding site. GOLD 5.0 was used for molecular docking since it was an excellent docking program. Gold Score was selected as the score function; number of dockings was set as 30; and the other parameters were set as default. The docking results were shown in Fig. 1b and c.

### Antibody and reagents

The primary antibodies VEGF-D, LYVE-1, β-actin were purchased from Santa Cruz Biotechnology (Santa Cruz, CA); VEGFR-3/phospho-VEGFR-3, JNK phospho-JNK, Bcl-2 and Bax antibodies were purchased from Cell Signaling Laboratories (Beverly, MA). The terminal deoxynucleotidyl transferase mediated nick-end labeling (TUNEL) assay kit was purchased from Promaga Company (Madison, WI). DMSO, Tween-20, Gelatin and CMC-Na were purchased from Sigma Chemical Co, (St. Louis, MO); Matrigel was purchased from BD Pharmingen (La Jolla, CA).

### Cell culture

Human lymphatic endothelial cells (HLECs) were purchased from the ScienCell™ Research Laboratories and cultured in endothelial cell medium (ECM) containing 5 % fetal bovine serum (FBS), 1 % endothelial cell growth supplement (ECGS), 100 IU/mL penicillin and 100 μg/mL streptomycin. Lewis lung carcinoma LL/2 cells were obtained from American Type Culture Collection (ATCC) and cultured in DMEM containing 10 % FBS, 100 IU/mL penicillin and 100 μg/mL streptomycin. Subcultures were performed with trypsin-EDTA. All cells were incubated in an atmosphere of 5 % CO_2_ at 37 °C.

### The establishment of VEGF-D high-expressing Lewis lung cell lines

The transfected murine LL/2 Lewis lung carcinoma cells with the pcDNA3.1 (+) expression vector containing mouse VEGF-D (VEGF-D-LL/2 cells) or pcDNA3.1 (vector alone) were transfected as reported before obtained in our laboratory [20–22]. The cell lines were maintained in an atmosphere of 5 % CO_2_ at 37 °C in cell culture medium DMEM supplemented with 10 % fetal bovine serum. Western blot analysis was performed to analyze the expression of recombinant VEGF-D of these cells.

### Cell viability assay

Cell viability assays were performed using CCK-8 kit [23]. Briefly, VEGF-D-LL/2 cells, parental LL/2 cells and HLECs were treated with various concentrations of YL529 in 96-well culture plates for 48 h. CCK-8 was added to the cells and the plate was incubated for an additional 2–4 h. The optical density (OD) was then measured at 450 nm using a Spectra MAX M5 microplate spectrophotometer (Molecular Devices, Sunnyvale, CA).

### Scratch-induced migration assay

The anti-metastasis effect of YL529 was determined using a scratch-induced cell migration assay *in vitro* [24, 25]. Briefly, 1 × 10^5^ HLECs were plated in 6 well plates and synchronized by serum-free ECM medium for 8 h, then used a micropipette tip to create a 2 mm-wide linear gap. Cells were washed to remove non-adherent cells and further incubated with fresh ECM medium containing or lacking YL529. An inverted microscope (Carl Zeiss, Germany) was used to photograph after YL529 treatment for another 48 h.

### Transwell migration assay

Transwell migration assay was adopted to indicate the anti-metastasis effect of YL529 [26]. Briefly, the transwell were pre-coated with 25 % Matrigel Matrix containing growth factors (BD Biosciences, Bedford, MA) for 30 min at 37 °C. And then the bottom chambers were filled with 600 μl ECM medium and the top chambers were seeded with 4 × 10^4^ cells HLECs (100 μl/well). The top and bottom chambers were incubated for another 24 h. Then cells on the top surface of the membrane were scraped with a cotton swab. Cells on the bottom side of the membrane were fixed with 4 % paraformaldehyde and stained with 0.1 % crystal violet (Sigma-Aldrich, USA). An inverted microscope (Zeiss, Axiovert 200, Germany) was used to obtain the images and the invading cells were quantified.

### Tube formation assay

The antilymphogenic effects of YL529 were analyzed *in vitro* using a tube-formation assay in HLECs. Briefly, 96-well plate was pre-incubated with 100 μl per well of Matrigel Matrix at 37 °C for 30 min, HLECs were seeded at a density of 3 × 10^4^ cells per well in ECM medium. And after cultured in the presence or absence of designed concentrations of YL529 on Matrigel Matrix at 37 °C another 6 h, tube formation by endothelial cells was evaluated and photographed under an inverse microscope (Zeiss, Axiovert 200, Germany).

### Western blot analysis

Standard western blot analysis was performed [23]. Briefly, Cell lysates were washed with phosphate buffered saline (PBS) and lysed in RIPA (radioimmunoprecipitation assay) buffer. Then lysates were centrifuged at 12000 g for 30 min at 4 °C. The Bio-Rad Protein Assay kit (Bio-Rad Laboratories) was used to determine the samples protein concentration according to the manufacturers’ recommendations. The lysates were dissolved in 5 × SDS sample buffer and denatured, then subjected to 6 % to 12 % SDS-PAGE (sodium dodecyl sulfate polyacrylamide gel electrophoresis) according to molecular weight and transferred onto PVDF (polyvinylidene fluoride) (Bio-Rad, Hercules, CA) membranes. Membranes were blocked for 1 h in 5 % dried milk in TBS/T at room temperature and incubated overnight at 4 °C with the primary antibodies and horseradish peroxidase-conjugated secondary antibodies. Protein bands were visualized with enhanced chemiluminescent substrate (Amersham Biosciences Corp., Piscataway, NJ).

### Pharmacokinetic characteristics analyses of YL529 in mice

C57 BL/6 mice (*n* = 3 per time point) were administered 150 mg/kg YL529 orally. Blood samples of the mice were collected at appropriate intervals and the plasma concentration of YL529 was analyzed by HPLC (Waters, MA, USA). The pharmacokinetic characteristics and parameters were analyzed using Pharmacokinetic Software of Drug and Statistics (DAS, edited and published by the Mathematical Pharmacology Professional Committee of China, Shanghai, China).

### Effect of YL529 on lymph metastasis in mice syngeneic models

Seven-week-old female C57 BL/6 mice (Beijing animal center, Beijing, China) were used. VEGE-D-LL/2 cells (1 × 10^6^) were injected intramuscularly and subcutaneously in hind limb of mice. The mice were randomized into 5 groups (*n* = 10 per groups) and orally administered 37.5, 75 and 150 mg/kg/day YL529, vehicle and saline alone (N.S.), respectively for 14 days when tumors became visible the 10^th^ day after cells were implanted. Tumor growth and mice weight was monitored every 3 days. When animals were sacrificed with CO_2_ gas at the end of drug administration, tumor tissues, lung organs and local lymph nodes were removed, weighted and calculated by manual. Tumor size was determined by measuring the largest and perpendicular diameters every 3 days, tumor volume was calculated using the formula: volume (mm^3^) = 0.5 × length × width^2^. In addition, the same subcutaneously tumor models were used to monitor the survival time of experimental mice after drug administration. All animals experiments performed were in accordance with the guidelines of the institute’s Animal Care and Use Committee of Sichuan University (Chengdu, China).

### Histological analysis for tumor tissue

Tumor tissues from vehicle and 150 mg/kg YL529 groups were fixed in 4 % paraformaldehyde, dehydrated and embedded in paraffin [27]. Sections of these tissues were subsequently incubated with LYVE-1 and corresponding second antibodies and visualized using peroxidase-DAB. TUNEL staining was also performed for fixed tissues (Promega, USA). Quantification was done as described [28]. Briefly, the microvessel counting was done in representative 200× fields or three high-power (400×) fields of the highest vascular density.

### Safety profile of YL529 *in vivo*

To evaluate the safety profile of YL529 (150 mg/kg) *in vivo*, we have observed the gross measures such as weight loss, life span, behavior and feedings. Moreover, we have also investigated the mortality and clinical signs of C57BL/6 mice throughout oral administration period. In addition, blood samples and heart, liver, spleen, lung, and kidney tissues of mice were collected; the histopathological, serum biochemistry and hematological analysis were done to observe the possible pathological changes. In addition, we also evaluated the safety profiles of YL529 after oral administration with high dose (6000 mg/kg) for 14 days.

### Statistical analysis

Data was expressed as mean ± SD/SEM. SPSS (SPSS, IL, USA) is used for statistical analysis. Statistical differences were considered significant when *p* < 0.05. Survival curves were constructed according to the Kaplan-Meier method, and the survivals were compared by means of the log-rank test.

## Results

### Molecular modeling and kinase inhibition profile of YL529

The novel multi-kinase small-molecule inhibitor YL529 have been identified by the computer-aided drug design (CADD), chemical synthesis and high-throughput screening (HTS) methods in our lab, and *in vitro* kinase binding assay showed that YL529 inhibited VEGFR2 activity at 10 μM,and YL529 significantly inhibited VEGFR-3 activity by 97 % at the same concentration [18]. In this study, we used computer simulation and computer-based molecular docking methods to further explore the interaction modes of YL529 with the kinase domain of VEGFR-3, and Fig. 1 depicts a possible binding configuration of YL529 with VEGFR-3. Based on these studies, we can see that the N-methylpicolinamide of YL529 forms strong hydrogen-bond interactions with the CYS930 residue in the hinge region of VEGFR-3. The N atom in amide moiety of YL529 forms another important hydrogen-bond interaction with the GLU896 in the DFG region of VEGFR-3. Additionally, YL529 also forms pi-related interactions with ASP1055, LYS879 and VAL859, consistent with what we have previously observed in YL529-VEGFR-2 interaction [18].

### YL529 inhibits proliferation, migration, invasion, and tube formation of HLECs *in vitro*

CCK-8 assay was used to evaluate the potential proliferation inhibition effect of HLECs after treated with YL529 or vehicle for 48 h. Our data showed that YL529 inhibited the proliferation of HLECs, with IC50 value of 5.5 μM.

**Fig. 2:**
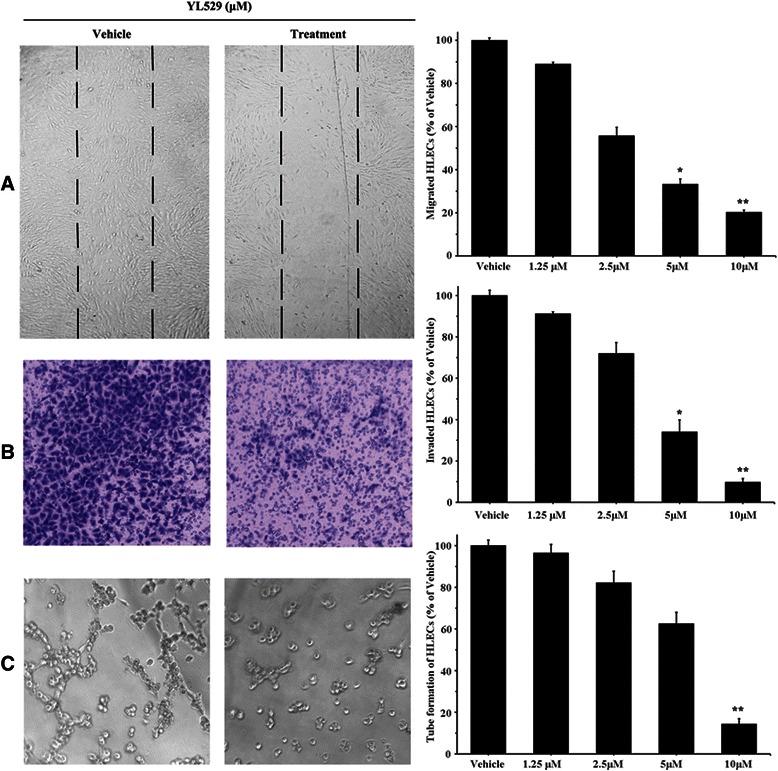
The effects of YL529 on HLECs. **a** Wound assay. Cells were wounded and migrated cells were quantified by manual counting; **b** Transwells assay. Invaded cells were stained and quantified; **c** Tube formation assay. 100×, Mean ± SEM, *n* = 3, *p < 0.05, **p < 0.01, ***p < 0.001

We next examined the effects of YL529 on HLECs migration using a wound healing migration assay [19]. The results showed that YL529 markedly decreased the number of migrating HLECs, as shown in Fig. 2a. At 2.5 μM, YL529 could inhibit the migration of cells by 44.3 %, and when the concentrations increased to 5 μM or 10 μM, the inhibition of cells migration was increased to 66.8 % or 79.8 %, respectively.

To further measure the effect of YL529 on HLECs invasion, we used a transwell assay and measured the number of HLECs that passed through a membrane barrier following treatment with various concentrations of YL529 [29]. As shown in Fig. 2b, compared with vehicle, 2.5 μM YL529 could inhibit the invasion of cells by 28.0 %, and when concentrations of YL529 increased to 5 μM or 10 μM, the inhibition of cell invasion was increased to 65.9 % or 90.2 %, respectively.

To further understand the mechanism of the anti-lymphogenic effect of YL529, the antilymphogenic tube formation effect of YL529 was analyzed with cultured HLECs *in vitro* [30]. As shown in Fig. 2c, YL529 treatment at 10 μM for 6 h strongly inhibited the formation of tube-like structures. Quantification showed that YL529 treatment inhibited tube formation by 85.7 %. In great contrast, HLECs without YL529 treatment spread and aligned with each other and formed a rich meshwork of branching capillary-like tubules within 6 h (Fig. 2c).

### YL529 inhibited the proliferation of VEGF-D-LL/2 cells

**Fig. 3:**
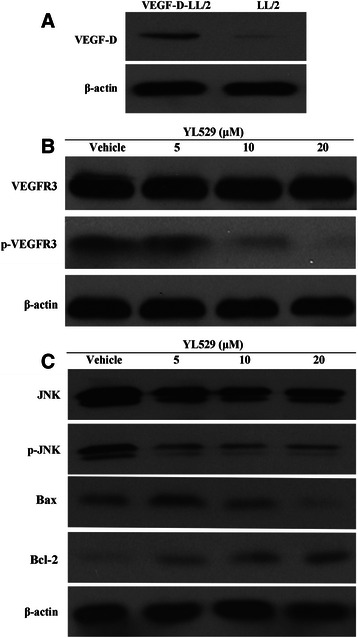
Western blotting to probe the molecular mechanisms of effects of YL529 *in vitro*. **a** p-VEGFR3 in HLECs was detected after YL529 treatment by western blotting analysis. **b** The overexpression of VEGF-D in VEGF-D-LL/2 cells was confirmed by western blotting analysis. The molecular weight of full-length VEGF-D was 21 KD. **c** VEGF-D-LL/2 cell were treated with YL529, p-JNK/JNK, Bax, Bcl-2 were analyzed by western blotting analysis

VEGF-D-LL/2 cells were previously established by transfection with the pcDNA3.1 (+) expression vector containing mouse VEGF-D [31]. Similar to the previous data from our group, this study uses a VEGF-D antibody that detects the mature form of VEGF-D, because the fully mature cleaved form of 21 kD has the greatest affinity for the receptors, and can bind and activate not only VEGFR-2 but also VEGFR-3. As shown in Fig. 3a, western blot analysis showed that the expression level of VEGF-D (21 kD) in VEGF-D-LL/2 cells was higher than the cells that were transfected with null-vectors (pcDNA-LL/2). This result confirmed that VEGF-D protein was constitutively up-regulated in VEGF-D-LL/2 cells. When VEGF-D-LL/2 cells and parental LL/2 cells were treated with YL529 or vehicle for 48 h, the calculated corresponding IC50 value was 7.2 μM and 9.6 μM, respectively. And we found that YL529 potently inhibited the proliferation of VEGF-D-LL/2 cells,

### Potential molecular mechanism of the effects of YL529 on HLECs and VEGF-D-LL/2 cells

To gain in-depth insight into the molecular mechanism of anti-lymphangiogenesis effects of YL529, the expression levels of VEGFR-3/p-VEGFR-3 in HLECs were analyzed by western blotting *in vitro*. As shown in Fig. 3b, in HLECs, YL529 potently inhibited the phosphorylation of VEGFR-3 after 2 h of treatment, even though no change of the total expression of VEGFR-3 was observed. This data may provide a molecular mechanism of the previously observed specific effects of YL529 on HLECs.

Moreover, as shown in Fig. 3c, YL529 decreased the expression level of p-JNK and Bax while increased the expression level of Bcl-2 in VEGF-D-LL/2 cells, which may explain the observed effects of YL529 on VEGF-D-LL/2 cells.

### Pharmacokinetics of YL529 *in vivo*

To determine the pharmacokinetic characteristics of YL529 in mice, C57 BL/6 mice were treated with YL529 orally and the key pharmacokinetic parameters of YL529 were summarized in Table 1, i.e., after oral administration of YL529 at 150 mg/kg, the peak plasma concentration (Cmax) was 18.03 μg/ml, the time-to-peak concentration (Tmax) was 2 h, the half-life (t1/2z) was 4.11 h and the AUC_0→∞_ was 170.95 mg/L · h, which suggested that the oral absorption and bioavailability and the pharmacokinetics of YL529 in mice are highly desirable.

**Table 1:** Pharmacokinetic parameters of YL529 after oral administration with single dose of 150 mg/kg in mice

Pharmacokinetic parameter	Value
AUC(0-∞)(mg/L*h)	170.95
AUC(0-t)(mg/L*h)	167.32
t1/2β(h)	6.03
T1/2α(h)	1.39
t1/2z(h)	4.11
Cmax(mg/L)	18.03
Tmax(h)	2.00

Data were expressed as the mean ± SD compared with vehicle. SPSS (SPSS, IL) software was used for statistical analysis (*n* = 3; *p < 0.05)

### YL529 inhibited tumor growth in VEGF-D-LL/2 tumors syngeneic models and extend the life span of affected mice

Based on the safety profile and the *in vivo* pharmacokinetics of YL529, chronic oral administration of YL529 at daily dosages of 37.5 ~ 150 mg/kg/day was chosen to treat the mice with VEGF-D-LL/2 tumors (syngeneic s.c. and muscle models). We found that YL529 inhibited tumor growth in dose-dependent manner in both models.

**Fig. 4:**
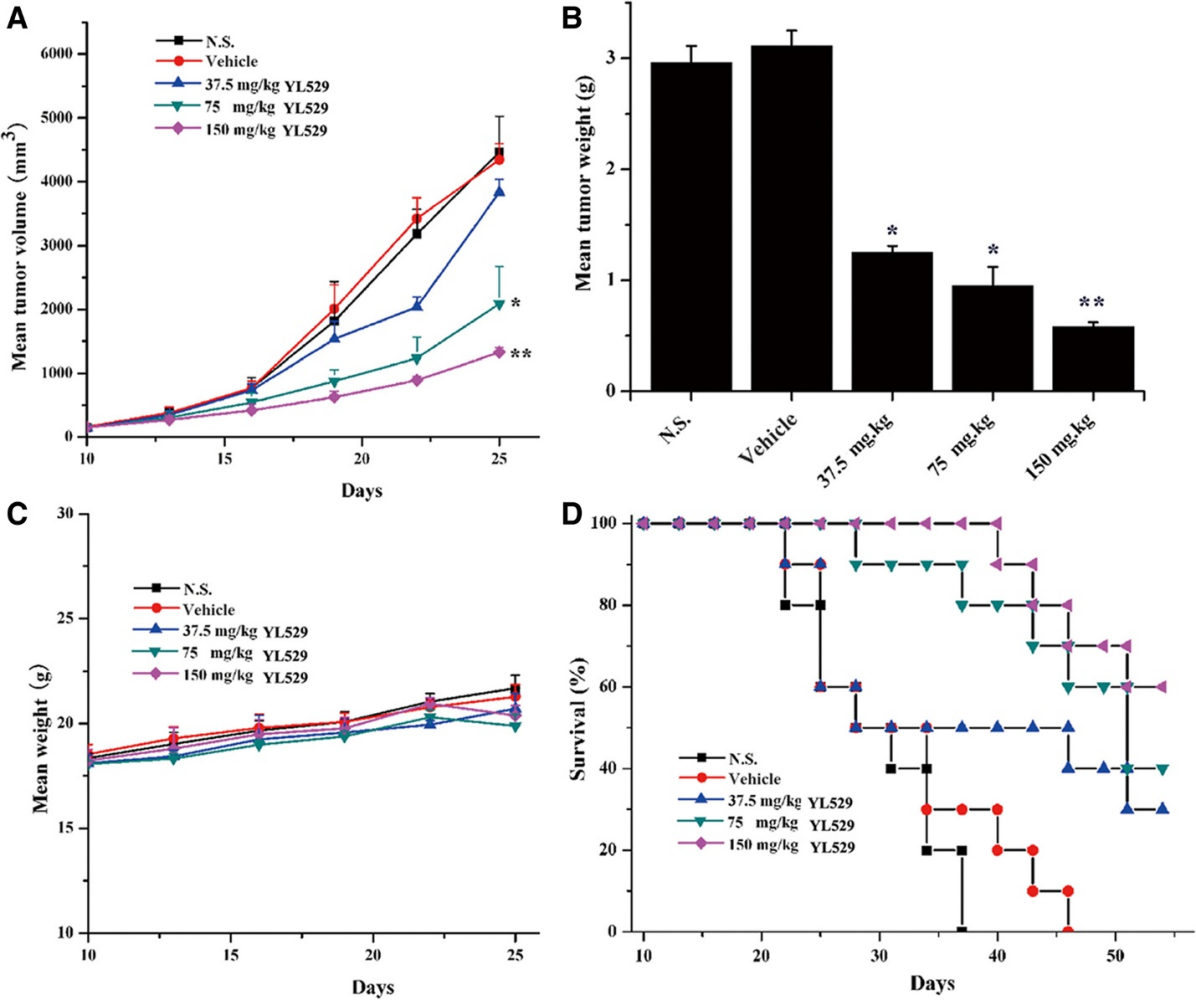
Effect of YL529 on tumor growth and survival in s. c. model. The mice bearing tumors subcutaneously were treated with YL529 or controls orally. **a** YL529 resulting in significant tumor growth inhibition; **b** Recovered tumor weights were significantly smaller in YL529 treated animals; **c** There was no significant difference of body weight after YL529 treated; **d** A significant increase in survival in YL529 treated mice was observed (Log-rank). Mean ± SEM, *n* = 10, *p < 0.05, **p < 0.01

Specifically, in s.c. model, as shown in Fig. 4a, there was a remarkable tumor volume reduction (66.22 %), compared with the vehicle-treated group, and the final tumor weights showed similar pattern (Fig. 4b), while there was no loss of body weight at this dosage (Fig. 4c). Furthermore, survival experiment *in vivo* showed that six out of ten animals in N.S. and vehicle groups died at 29 days (Mice were regarded as sacrificed when the diameter of tumors reached about 20 mm), while in YL529 treated groups, the life span of the mice have been significantly extended (Fig. 4d, *p < 0.05, by log-rank test), i.e., 90 % of mice survived to 40 days and 60 % of mice in 150 mg/kg group survived to 54 days when we finished the survival observation.

**Fig. 5:**
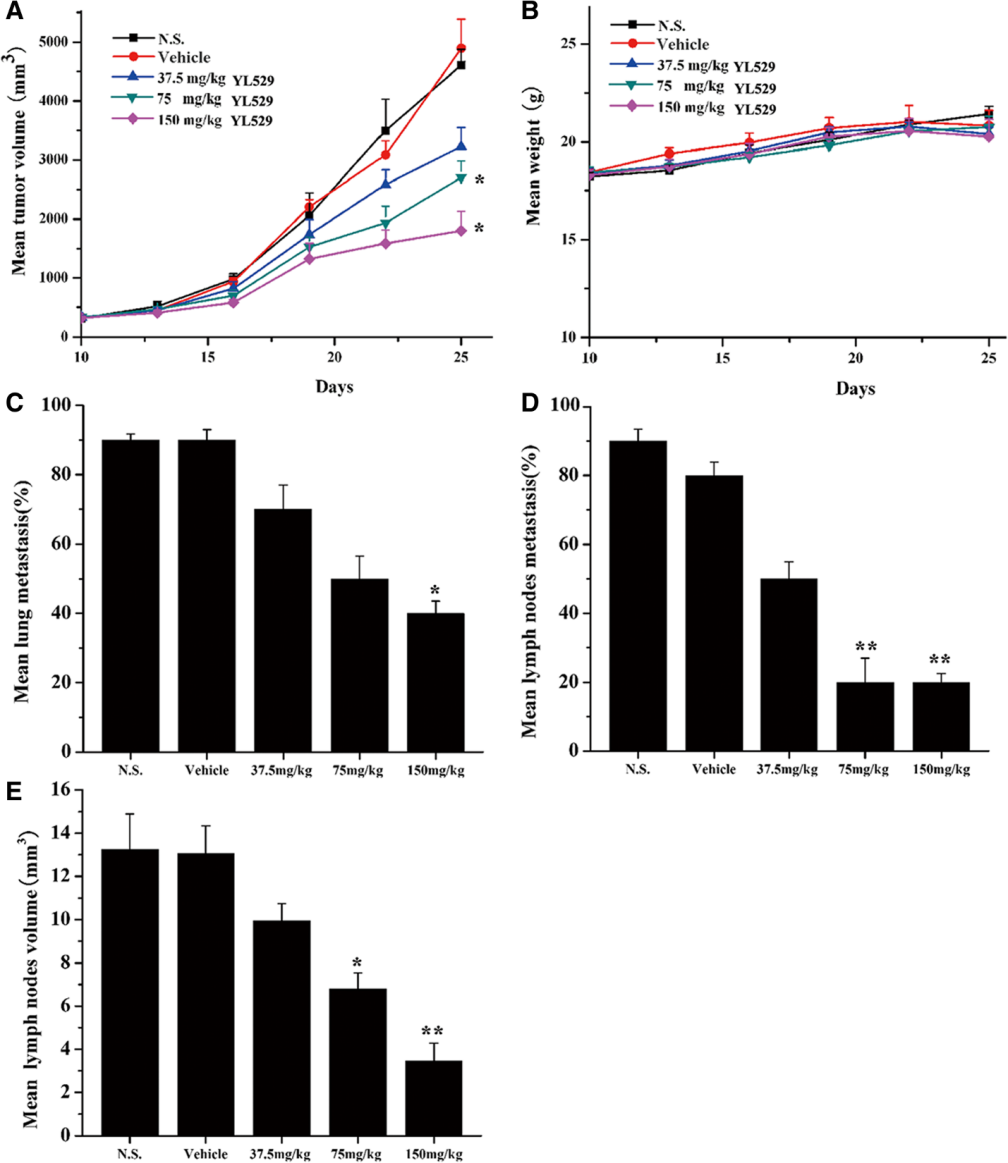
Effects of YL529 on tumor growth, lung and lymph node metastasis in muscle model. The muscle metastasis models were treated with YL529 or controls orally. **a** YL529 resulting in significant tumor growth inhibition; **b** There was no significant difference of body weight after YL529 treated; **c** and **d** Percentage of mice with lung and lymph node metastasis; **e** Mean volume of auxiliary lymph nodes were harvested from mice. Mean ± SEM, *n* = 10, *p < 0.05, **p < 0.01

Similar results were observed in the muscle model (Fig. 5a) (*p < 0.05). Moreover, it is worthy to stress that YL529 also significantly reduced the number of mice with lung metastasis and inguinal lymph node metastasis in the tumor muscle model, while there was no loss of body weight at this dosage at the end of drug administration (Fig. 5b). similarly, as shown in Fig. 5c, d and e (*p < 0.05, **p < 0.01), the percent of mice with local lymph node metastasis in vehicle group could reach 80 % ~ 90 %, while reaching only 50 % in 37.5 mg/kg group and 20 % in 150 mg/kg group, respectively.

#### Safety profile of YL529 in a preclinical study

Currently, side effects are the prevalent shortcomings of anticancer drugs. In our current study, mice treated with YL529 at the daily dosage of 150 mg/kg for 18 days showed no obvious body weight loss or tissue damage, which is consistent with our previous reported [18]. Furthermore, we further evaluated the toxicity in SD rats treated by YL529 at the daily dosage of 6000 mg/kg for 14 days, and we found that there were no obvious weight changes of major organs (heart, liver, spleen, lung, kidney and brain) (Table 2), or obvious pathological damage, or other adverse effects (Data not shown).

**Table 2:** The organs parameters of YL529 after oral administration 6000 mg/kg YL529 with a single dose in SD rats

Index	Vehicle	YL529	Vehicle	YL529
	Female(♀)	Female (♀)	Male (♂)	Male (♂)
Heart (%)	0.41 ± 0.08	0.42 ± 0.09	0.43 ± 0.05	0.42 ± 0.06
Liver (%)	3.11 ± 0.10	3.13 ± 0.09	3.09 ± 0.10	3.09 ± 0.13
Spleen (%)	0.23 ± 0.03	0.25 ± 0.03	0.23 ± 0.09	0.24 ± 0.04
Lung (%)	0.43 ± 0.05	0.43 ± 0.06	0.44 ± 0.11	0.42 ± 0.09
Kidney (%)	0.37 ± 0.06	0.38 ± 0.09	0.36 ± 0.08	0.38 ± 0.09

Data were expressed as the mean ± SD compared with vehicle. SPSS (SPSS, IL) software was used for statistical analysis (*n* = 10; *p < 0.05)

### YL529 inhibited lymphangiogenesis in tumor tissues in additional to induce tumor cell apoptosis *in vivo*

**Fig. 6:**
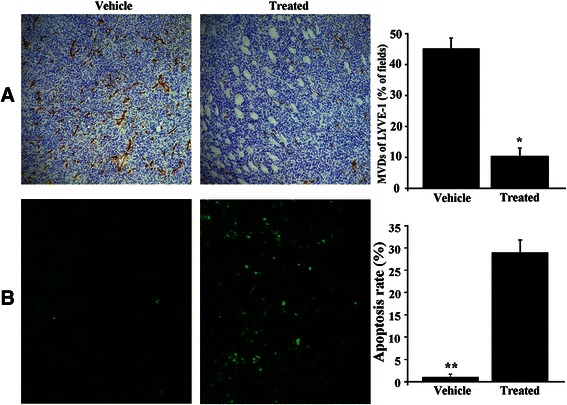
Immunohistochemical analysis of LYVE-1 and TUNEL assay *in vivo*. Sections from vehicle group and 150 mg/kg YL529-treated tumor were collected. **(a)** LYVE-1 and **(b)** TUNEL were detected in the VEGF-D-LL/2 tumor model. Quantitative of the mean LYVE-1 and TUNEL-positive area counted at × 200. Mean ± SEM, 200×, *n* = 6, *p < 0.05, **p < 0.01

The immunohistochemical analysis and TUNEL apoptosis assays and LYVE-1 staining were performed to directly evaluate whether YL529 could inhibit the tumor lymphangiogenesis and induce tumor cell apoptosis *in vivo*. As shown in Fig. 6a, YL529 remarkably decreased the amount of lymphangiogenesis in tumor tissues at 150 mg/kg for 14 days in VEGF-D-LL/2 tumor syngeneic model, as indicated by the LYVE-1 staining (34.71 % higher than the vehicle group in VEGF-D-LL/2 muscle model). Moreover, TUNEL assay found that there were a dramatic higher number of apoptotic tumor cells in the treated group (20-fold increase vs. vehicle), in s.c. model (Fig. 6b).

## Discussion

Metastasis, particularly via the lymphatic system, is a common prognostic factor and critical process in the spread of solid cancer [2]. The VEGF/VEGFR pathway has been widely studied because VEGFR expression is strongly correlated with tumor metastasis progression and poor prognosis. Therefore, this pathway has been pursued as a therapeutic strategy for inhibition of lymphangiogenesis and metastatic in tumors,and some animal studies have shown that the expression of VEGF-D promotes lymphangiogenesis and metastatic spread of tumor cells via lymphatic in papillary thyroid carcinoma, lung cancer and gastric cancer [32–34]. However, most studies are based on cultured tumor cells. Little *in vivo* research has been done to correlate the high VEGF-D levels and lymph node metastasis in lung carcinoma, thus, it remains unclear whether expression of VEGF-D in culture accurately represents the *in vivo* situation.

Furthermore, since VEGF-D plays a remarkable role in up-regulating lymphangiogenesis and regional lymph node metastasis, other researchers in our group has established the mouse lymph node metastasis model by transfecting high expression VEGF-D into LL/2 Lewis lung carcinoma cells, and found that VEGF-D mainly bind to VEGFR-3 in lymphatic endothelial cells *in vivo* [31], and further study demonstrated that VEGFR-3 contributes to autocrine effects through VEGF-D-LL/2 cells [20].

YL529 was developed as a potential multikinase anticancer agent in our laboratory using CADD, HTS, *de novo* synthesis and has been reported to have a variety of pharmacological effects such as inhibiting tumor growth via antiangiogenesis and anti-proliferation in our previously study, especially it could block the activities of human umbilical vein endothelial cells (HUVEC) stimulated with VEGF_165_ previously [18]. And some researchers have reported that VEGFR-2 can also be expressed on lymphatic endothelial cells, while its expression levels were less than VEGFR-3 in lymphatic ECs, because VEGFR2 is the major effector of angiogenesis and regulates blood vessel growth of vascular ECs, not lymphatic ECs [35–37]. However, it is still possible that some of its inhibitory activities can be assigned to VEGFR-2, because YL529 is a multikinases inhibitor.

To further address some caveats, we determined that YL529 inhibits the proliferation and migration of high-expressing VEGF-D Lewis lung carcinoma LL/2 cells. Similar effects of YL529 were also observed in HLECs, which endogenously express high level of VEGFR-3. Consistent with this idea, we found that the anti-proliferation effect of YL529 was dependent on high VEGF-D expression, since YL529 has no as obvious effect on the parental LL/2 cells, which has low level of endogenous VEGF-D expression (Data not shown). Interestingly, high expression of VEGF-D has been found to correlate with the up-regulation of Fra-1, a member of the AP-1 family of transcription factors [38]. And AP-1 interacts with Fos and Jun protein family members, and the latter is regulated by ERK1/2 and JNKs, respectively.

Mechanistically, our result showed that YL529 downregulation of p-VEGFR-3 in HLECs cell in a dose-dependent manner, which is consistent with the computer-aided drug design (CADD) and molecular reverse docking results. Furthermore, we found that YL529 also inhibited the phosphorylation of JNK1/2, while the total protein expression of JNK1/2 was not changed. Along this line, YL529 can also decrease the expression level of Bax and upregulate Bcl-2, which supported the idea of a potential pro-apoptotic effect of YL529. Taken together, it is reasonable to propose that YL529 could not only down-regulated the levels of p-VEGFR-3, but also blocked the VEGFR-3 signaling pathway by interfering with the expression levels of p-JNK and induced apoptosis via down-regulating Bax and up-regulating Bcl-2.

These results are further supported by the fact that YL529 inhibited the tumor-associated lymphangiogenesis and metastasis in mice harboring VEGF-D-LL/2 Lewis carcinoma *in vivo*, and by immunohistochemical staining showed that YL529-treated tumors displayed less LYVE-1 positive vessels compared with vehicle.

Our data has confirmed that YL529 exhibits the characteristics of blocking tumor metastasis at several different time points in the process of tumor lymphangiogenesis for the first time: (I) YL529 inhibits proliferation and induces apoptosis of in high expression VEGF-D tumor cells; (II) YL529 downregulates the expression of VEGFR-3 on HLECs and blockades the VEGFR-3 signaling pathways by interfering with the activation of JNK1/2; (III) YL529 interrupts and inhibits HLECs proliferation, invasion and tube formation; (IV) YL529 inhibits the tumor-associated lymphangiogenesis; (V) YL529 suppresses tumor cell migration and induces apoptosis. Moreover, there is a closely associated between lymph node metastasis and VEGFR-3 expression in several lewis lung carcinoma models. The binding of VEGF-D and VEGFR-3 could promote migration and proliferation of HLECs through JNKs signaling pathways. So blockade of the VEGFR-3 pathways could efficiently inhibit tumor lymphangiogenesis and metastasis. These results support the hypothesis that the therapeutic effect of YL529 on tumor lymphangiogenesis and metastasis could be attributed to direct inhibition of lymphangiogenesis in lung tumor models induced by VEGF-D and through down-regulation of VEGFR-3.

## Conclusions

**Fig. 7:**
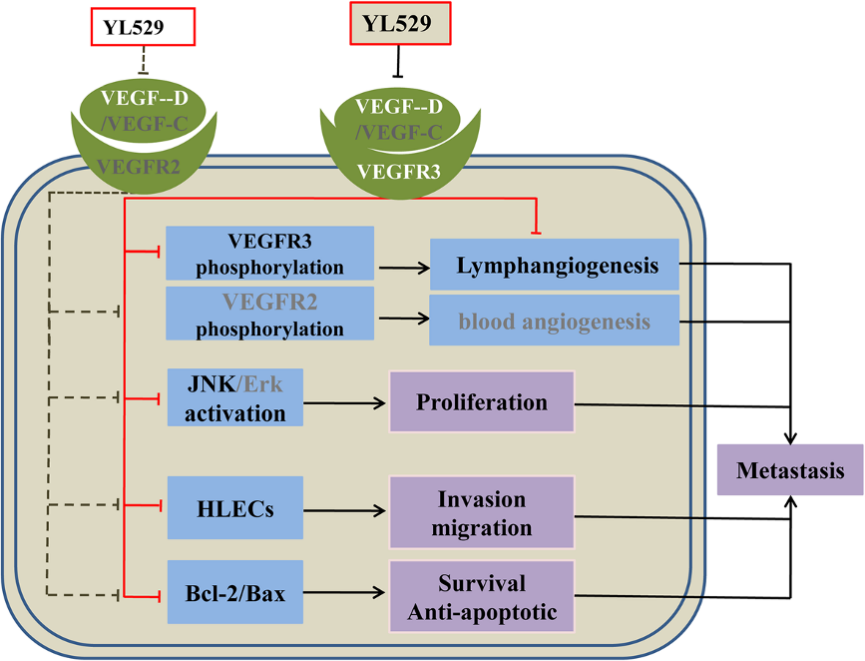
The proposed underlying mechanisms of YL529

In conclusion, our results indicated that YL529 significantly inhibited the tumor-associated lymphangiogenesis and metastasis induced by VEGF-D in the established VEGF-D over-expressing Lewis lung carcinoma model (Fig. 7). Tumor growth was significantly retarded and prolonged life span was observed after YL529 treatment in tumor-bearing mice. We have demonstrated for the first time that YL529 suppresses tumor lymphangiogenesis and lymphatic metastasis by down-regulation of VEGF-D in VEGF-D-LL/2 cancer cells and inhibiting neogenesis and tube formation of lymphatic endothelial cells directly through the VEGFR-3 pathway. Our findings suggest that YL529 may be an attractive agent against lymphangiogenesis and metastasis. Moreover, YL529 has a novel chemical structure that is different from VEGFR-3 inhibitors in clinical use. In addition, YL529 was well tolerated by the host animal in therapeutically beneficial doses, making it a promised candidate for phase I clinical trials as an antilymphatic metastasis drug.

## Abbreviations

HLECs: Human lymphatic endothelial cells
VEGF: Vascular endothelial growth factor
VEGF-C/D: Vascular endothelial growth factor-C/D
HUVECs: Human umbilical vein endothelial cells
HPLC: High performance liquid chromatography
NMR: Nuclear magnetic resonance
DMSO: Dimethyl-sulphoxide
CMC-Na: Carboxymethylcellulose
ECM: Endothelial cell medium
FBS: Fetal bovine serum
ATCC: American type culture collection
OD: Optical density
PBS: Phosphate buffered saline
RIPA: Radioimmunoprecipitation assay
PVDF: Polyvinylidene fluoride
SDS-PAGE: Sodium dodecyl sulfate polyacrylamide gel electrophoresis
Cmax: The peak plasma concentration
Tmax: The time-to-peak concentration
t1/2z: The half-life
TUNEL: Terminal deoxynucleotidyl transferase mediated nick-end labeling
